# A Thematic Analysis of Motivators and Barriers to Antimicrobial Resistance Interventions With Farmers and Animal Health Professionals in Nigeria

**DOI:** 10.1155/vmi/8043291

**Published:** 2025-10-21

**Authors:** Alice B. J. E. Jacobsen, Jane Ogden, Aliyu Wakawa, Abel B. Ekiri

**Affiliations:** ^1^Department of Comparative Biomedical Sciences, School of Veterinary Medicine, University of Surrey, Guildford, UK; ^2^School of Psychology, University of Surrey, Guildford, UK; ^3^Department of Veterinary Medicine, Faculty of Veterinary Medicine, Ahmadu Bello University, Zaria, Kaduna, Nigeria

## Abstract

Antimicrobial use (AMU) in animals, including poultry, can contribute to antimicrobial resistance (AMR) in humans. With a rising middle class and demand for meat in sub-Saharan Africa's second largest poultry producer, Nigeria, AMU in food-producing animals is predicted to continue rising. Interventions to reduce AMR and AMU are limited in low- and middle-income countries, including Nigeria. This study aimed to understand the current AMU practices, challenges and motivators and barriers to adopting AMR interventions in the Nigerian poultry sector. Qualitative semistructured interviews (*n* = 22) were conducted in Nigeria consisting of poultry farmers, animal health professionals and other related key players. Thematic analysis identified three themes surrounding barriers and challenges: (i) ‘issues of access' relating to time, money, laboratories and expertise, (ii) ‘lack of knowledge' due to lack of training and poor relationships between farmers and animal health professionals and (iii) ‘taking responsibility' with participants describing a lack of responsibility from both the government and other animal health professionals, para-professionals and farmers and how social responsibility was key to motivating people. Overall, the results from this study highlighted the wide range of barriers to engaging animal health professionals and farmers in AMR interventions and improving AMU practices in sub-Saharan Africa. If interventions are to succeed, they need to reflect a collaborative and multifaceted effort from all invested parties.

## 1. Introduction

Antimicrobial resistance (AMR) is a major concern for human and animal health globally. In 2019, sub-Saharan Africa (SSA) had the highest rate of deaths attributed to AMR, 50% higher than the global average [1]. The use of antimicrobials in livestock and humans has been linked to the AMR in humans.

Nigeria has the largest human population in Africa. With a growing middle class, like many other low- and middle-income countries (LMICs), the demand for meat is increasing [2]. Currently, Nigeria is among the top broiler producers in Africa, second to South Africa [3, 4]. The use of antibiotics in livestock, including poultry, for disease management is expected to continue increasing in parallel with the increased demand for animal proteins.

The anticipated increase in the use of antimicrobials in poultry and other livestock raises important public health concerns and highlights the need for interventions. The use of critically important antimicrobials (CIAs), antimicrobials considered essential for treating resistant and severe infections in humans, has been reported in animals in Nigeria. The use of antimicrobial drugs in livestock in Nigeria increased by 40.4%, with the majority classified as CIAs, including fluoroquinolones (26.5%) and beta-lactams/aminoglycosides (20.4%) for the period 2010–2012 [5, 6]. Furthermore, a study of Nigerian poultry farmers reported that 98% of the farmers administered antimicrobials for prophylaxis to day-old chicks, including CIAs [7].

A recent review by Jacobsen et al. [8] has investigated interventions aimed at reducing AMR, Antimicrobial use (AMU) and increasing antimicrobial stewardship (AMS) globally and reported that only 19% of interventions (*n* = 90) were conducted in LMICs, with only three conducted in SSA and none in Nigeria.

The limited data on AMR interventions in SSA, particularly Nigeria, highlight the need to understand why there are no clear interventions aimed at reducing the use of antimicrobials in the livestock sector, specifically in poultry, in Nigeria. As a first step, the understanding of the motivators and barriers to the implementation of AMR interventions may help to identify and tailor relevant interventions to the target populations of interest. Currently, little is known about the motivators and barriers to the implementation of AMR interventions in the poultry sector in Nigeria. However, there is some evidence from the poultry sector in other LMICs. For example, a study was conducted in Indonesia using semistructured interviews to understand the barriers to changing AMU practices in the poultry sector, targeting farmers and associated professions (including veterinarians, technical staff, academics and private companies) [9]. The identified barriers included a lack of understanding around AMU, financial pressures within the poultry sector and a lack of engagement with the government veterinary services [9].

Even though there is no evidence on barriers within the poultry sector in Nigeria, existing evidence from the human medical sector may transfer to the poultry sector. Such barriers include cost and unavailability of laboratory services, unavailability of guidelines, delays in result reporting [10] and a lack of continued medical education and pharmaceutical company marketing [11]. Aiming to understand the motivators and barriers more specifically within the poultry sector can help tailor interventions.

The aim of this study was to improve understanding of the motivators and barriers to the implementation of AMR interventions in the poultry production sector by animal health professionals (AHPs) (veterinarians and para-veterinarians), farmers and other related key players (agriculture ministry, veterinary laboratories and feed producers) in Nigeria. The knowledge gained can be used to identify, develop and tailor AMR interventions to the target populations of interest, including poultry farmers and veterinarians, and to inform further research on AMR interventions.

## 2. Methods

### 2.1. Study Design and Area

This qualitative study used reflective thematic analysis [12] with semistructured interviews. The study area comprised four states in Nigeria: Kaduna, Kano, Plateau and Oyo, selected to reflect the geographical and human population variation, as well as the distribution of poultry production, with the majority of poultry concentrated in the Southwest (Oyo).

#### 2.1.1. Participant Selection and Recruitment

The study participants were from Nigeria and included three groups: commercial poultry farmers (with a minimum of 300 commercial birds on the farm, either layers, broilers or breeders), AHPs (veterinarians and para-veterinarians) and other related key players (ORKPs) (individuals working in the agriculture ministry, pharmaceutical industry [importers, distributors and retailers], feed processing companies and veterinary diagnostic laboratories) (Table 1). The two key groups of interest were AHPs and farmers. The group other related key players was added to provide context to the interviews with AHPs and farmers.

Sample size considerations: A minimum of 12-13 interviews with poultry farmers and AHPs (veterinarians and para-veterinarians) was estimated to achieve saturation [13, 14]. The interviews conducted with other related professionals were not designed to reach saturation as they were intended to only provide context to the interviews with AHPs and farmers.

The recruitment of participants took place between October 2023 and March 2024. The participants were contacted by email by one of the co-authors, AW. To further increase the sample size, participants were encouraged to inform their colleagues about the study verbally and through the use of flyers describing the study. Participants were asked to contact the study team (ABJEJ), who scheduled and performed the online interviews via a virtual platform (Zoom).

Participation in the study was voluntary, and participants were able to withdraw at any point. There was no reimbursement or incentive provided to participants. Participant information and a consent form were provided online and were completed before participants were asked to arrange a date for online interviews.

#### 2.1.2. Setting

The interviews took place from October 2023 to March 2024. The data were collected online on Zoom, with the interviewer seated in a quiet, private environment. The online interviews allowed for the conduct of interviews across a wider geographical area of Nigeria and over a more extended period, which may not have been achieved through traditional face-to-face interviews.

#### 2.1.3. Data Collection

ABJEJ conducted the interviews. None of the participants were known to the interviewer (ABJEJ). Participants were informed at the start of the interview that the interviewer (ABJEJ) was a veterinarian with veterinary clinical knowledge and a One Health background. Participants were also informed that the study was part of ABJEJ's doctoral research.

A semistructured interview tool was developed and used as a guideline to conduct the interviews. The interview included primarily open-ended questions, and prompts were used to encourage elaboration and to ensure a rich and full dataset. The first part of the interview consisted of inductive questions intended to understand the participants' background, knowledge and opinions on motivators and barriers to interventions that aim to reduce AMU and increase AMS.

The second part was deductive and asked participants' thoughts on specific interventions and their feasibility within the target population. The interview tool, including the prompts and aim for questions, is provided in the supporting information (Supporting Information 1). The interviews lasted, on average, 25 min and were recorded (audio, video and live transcription).

### 2.2. Data Analysis

Data was analysed using the six steps of reflective thematic analysis [12, 15]. This involved the following: familiarisation with the dataset and performing of initial analytic observations; coding of the data and creating labels to annotate the features of the data addressing the research question; creating initial themes by searching for broader patterns in the codes; furthering the initial theme through development and revision; refining, defining and naming the themes; and writing up. ABJEJ took the lead in the analysis with discussion and input from JO and ABE. The data analysis was performed in NVivo R1.

### 2.3. Positionality

The data were collected by ABJEJ, who identifies as a woman, is White European and is a predoctoral researcher. The reflective process of coding and refining themes was discussed with ABE who identifies as a Black African man and JO who identifies as a White British woman. Both ABJEJ and ABE are veterinarians, while JO is an academic psychologist. To mitigate researcher bias and to encourage openness, all participants were reassured that the interview was anonymous, and some common ground was established by the interviewer identifying herself at the start of the interviews as someone who works within and understands the animal health sector.

## 3. Results

**Figure 1:**
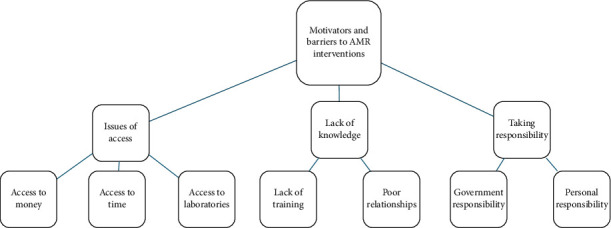
Thematic map of themes and subthemes identified through a reflective thematic analysis of the data.

A total of 22 qualitative semistructured interviews were conducted with poultry farmers, AHPs and other related key players (Supporting Information 2). Three themes were derived from the interview data: *issues of access, lack of knowledge, and taking responsibility.* The themes and subthemes are illustrated in the thematic map (Figure 1).

### 3.1. Theme 1: Issues of Access

Farmers, AHPs and ORKPs described barriers around access to creating and performing interventions to reduce AMU and AMR. These were primarily described as structural, including access to time, money, expertise and laboratories.

#### 3.1.1. Access to Time

Farmers often had other jobs alongside poultry farming. Some worked other jobs within the food animal production industry, while others worked in other sectors. This meant that farmers did not have time to be at their farms every day to check on their birds and farm workers. They also expressed having limited time to undertake or participate in continuous professional development.

*Well, one, most of the farmers are not full-time farmers, so they have other works they do. They have a nine to five work and then they attend to. Then sometimes the timing will not be convenient for them. (P.8) Farmer and Veterinarian*

*Have you attended any such courses [continued professional development]? (Int)*

*Yes. No, not really. I actually, I'm so, so busy with my work. (P.10) Farmer*

#### 3.1.2. Access to Money

Lack of monetary funds was also described as a barrier to engaging with AMR interventions. Lack of funds included funds to cover costs for feeding and raising birds and funds to pay for services provided by AHPs or other professional assistance. Farmers indicated that they were struggling financially to stay afloat and to keep farms running.

*Hmm. They can hardly make ends meet and I can count the number of farms around my area where about two or three of them have folded up because they can't cope. I'm basically doing it because of my love for it. (P.11) Farmer*

Other farmers expressed lack of money, resulting in not being able to update their farm biosecurity to the level they desired.

*I use borehole in the farm to get water, so I'm trying to look at how can I have a system where I minimise water storage. So, what I expect is immediately the water came out from the borehole it will go directly to the to the chicken without storing it somewhere. It will reduce the risks or contamination and any other things that will happen in between. (P.13) Farmer*

Farmers also expressed concerns about the economic implications of continuing to care for and feed slaughter-ready birds when observing the withdrawal period following treatment with antibiotics. The fear of economic loss leads farmers to slaughter birds before the withdrawal period is over.

*Did you talk to the butchers and the farmers about why they choose to slaughter while they're still on antibiotics? (Int)*

*Yes. I asked them why they do that, and I think most of them most of them the challenge is that is the economic implication. (P.18) Academic Researcher*

The lack of monetary funds also extended to research that could help to reduce the use of antibiotics.

*So, I just did experimented. I just kept the experimental birds, but I did not. I did not have the funding to do the field sequence. (P.17) Academic Researcher*

#### 3.1.3. Access to Laboratories

Most farmers and veterinarians expressed that they would like to use laboratories, but due to multiple factors, including the high cost, slow turnaround time and distance to laboratories, they were not used as much as they could be.

*And we also have few labs available here. It's because cost of that too. At the end of the day, we add it to the bill and the farmers are seriously trying to avoid cost. So, I believe that is what has exposed them into the use of antibiotics. (P.4) Veterinarian*

A slow turnaround time from submission of samples to receiving laboratory results meant that some farmers opted to use antibiotics in the interim, while some farmers trialled different antibiotics instead of following the advice of AHPs to wait for the laboratory results. The slow turnaround was also recognised by laboratory professionals.

*Yes, because here if you go for sensitivity, at least 24 h. Some up to 48 h, so the timing is also an issue, so it's on if a farmer is having so much mortality, and then you tell the farmer to wait for a day or two before commencing treatment. Yeah, most of them will not heed their advice. …Yeah. So, the timing, so the timing is also an issue. (P.8) Farmer and Veterinarian*

One laboratory was aware of and trying to combat the 48-h turnaround time.

*Because that time gap too is there when they come to the laboratory, and you tell them on in the next 48 h they will get the antibiotic they are going to use. That one is also a problem to them, you know? Wait. So, what's my own unit is trying to do is trying to see how the microbiology, you know, analysis and you know, look for, you know, looking for laboratory equipment that can help us. (P.20) Veterinary laboratory professional*

Not all AHPs, however, expressed concerns related to access and turnaround time for laboratory testing results. One AHP expressed having good access to labs in their area and described being happy with the 48-h turnaround time.

*Where I am that is not much of an issue [lab access]. Because we have one or two standard laboratories. Where we can take samples to within two days or two days the results will be emailed to us. (P.4) Veterinarian*

#### 3.1.4. Access to Expertise

Access to AHPs was reported as an issue, with some areas not having access to local AHPs.

*That is why if they have veterinarians readily available then they are very very happy you know. (P.16) Other Related Key Player (Pharmaceutical Industry)*

Furthermore, even when participants could access AHPs, several farmers and other related key players described difficulties in affording veterinary care.

*But a lot of them what they're doing now is just blind, you know, those people are unable to afford [inaudible] veterinarian. (P.16) Pharmaceutical Industry*

The inability to afford veterinarians (degree holder AHPs) meant that farmers might choose to use a para-professional (diploma or certificate holder AHPs) as they were likely cheaper. The para-professionals were also more likely to speak the local dialects of farmers and farmhands.

*Yeah, they speak the language much more and they [para-professionals] would build relationship with these farmers, and they might not charge, or they might not charge as much as normal active veterinarian. (P.7) Farmers and Veterinarian*

Many veterinarians expressed frustration with para-professionals who were viewed as not having appropriate animal health qualifications, referred to as ‘quacks', and described how farmers often chose to use para-professionals instead of a veterinarian.

*Another major problem we have here is we have a lot of quarks. That are not fully qualified in the field. Some are part of it. Some are just little knowledge… So, at the end of the day these people become they now call themselves doctors and they impersonate the role. (P.4) Veterinarian*

In summary, one key barrier to the use of interventions to reduce antibiotic use was reduced access, particularly related to structural issues, including lack of time, funding and access to AHPs and laboratories.

### 3.2. Theme 2: Lack of Knowledge

Participants described having knowledge as key to the use of interventions to change antibiotic use. For some, the focus was on a lack of knowledge on antibiotic withdrawal periods, while some expressed concerns about the dangers of too much knowledge. This was highlighted as a product of a lack of training and poor relationships between the different parties involved.

#### 3.2.1. Lack of Training

The participants from different targeted groups (farmers, AHPs, etc.) described how a lack of knowledge on AMR and AMU stemmed from a lack of training relating to AMR and AMU.

*…that level of knowledge is simply lacking in veterinary, right. So having that just to provide guidance for clinicians will go a long way in ensuring that the people really do not, you know, resort to last resort, antibiotics. (P.3) Veterinarian*

Furthermore, a lack of training around how and when to engage with laboratories was also highlighted.

*Actually, in my view, I think is the training actually right from school. I guess you know there's little or not much emphasis on the role of the lab in their decision-making. Before you realise that actually they don't really care much about it. So, one can say that I don't know. (P.19) Veterinary Laboratory Staff*

The participants also described how para-professionals, who are diploma or certificate holders, did not have sufficient knowledge to make clinical management decisions or decisions on appropriate use of antibiotics.

*What the antibiotic is, is targeting, they [para-professionals] don't really have that knowledge. All they know is that they have seen a doctor use it. (P.4) Veterinarian*

A concern of farmers lacking knowledge of the consequences of inappropriate use of antibiotic use was highlighted.

One area that was frequently described was the lack of knowledge about withdrawal periods for antibiotics. When farmers have sick birds that have been given antibiotics but are not improving, the birds are slaughtered and sold before the withdrawal period is over.

*…there's no withdrawal period. They can give these week later, next week selling. And those buying are not are not aware of what is going or perhaps they don't know the significance of what is going on. (P.17) Academic Researcher*

While most participants described that the lack of knowledge of AMR was a problem, some farmers were deemed to be more knowledgeable.

While more knowledge was generally regarded as positive, one AHP described how farmers having more knowledge could be problematic and may promote self-prescribing behaviour of antibiotics.

*… but the problem here is the farmers being more informed about diseases and antibiotics, has turned out to be one of the biggest problem. Because they feel they have this sense of they know it, a lot of them can just take off their phone and then Google up something. With the information they get on Google, they just go straight ahead to administer drugs. (P.4) Veterinarian*

#### 3.2.2. Poor Relationships

While the lack of knowledge was deemed to result from the absence of training, it was also seen as resulting from poor relationships between farmers and AHPs. In particular, AHPs described how farmers were often unwilling to tell them the truth about the antibiotics administered to their birds.

*So, because that they don't open up. Give us full information. So, I'd like it before taking any decision they'd reach out to us. (P.4) Veterinarian*

*You know, at times they might not really tell you whether or not they gave their animals antibiotic. We'll just bring it to you for treatment. (P.6) Veterinarian*

One laboratory worker expressed how they felt it was out of fear of reprimand that farmers did not want to tell the truth.

*So, they will not want me to scold them or to tell them that all they have done is bad. So, they want to hide. They tell you that they have not done anything. (P.20) Veterinary Laboratory Staff*

The lack of good relationships between farmers and AHPs meant that some AHPs were excluded by farmers from the process of deciding on and using antibiotics.

*And naturally, the other farmer begins to make prescriptions begin, give advice, like “OK, use this drug, use that drug, do this” at the end of the day, the veterinarian is left out. (P.4) Veterinarian*

*And he himself [the farmer], now he's the doctor of himself. He goes to the market to buy any product for his farm. (P.4) Veterinarian*

The unwillingness of farmers to engage AHPs extended to some farmers discontinuing the services of the AHPs on their farms.

*I currently have a farmer that I've been talking to recently, he has, he has a sizeable farm. He has fired his, his veterinarians and everybody working in his farm because he was not getting results from them. (P.4) Veterinarian*

AHPs found that even if farmers came to them about their sick birds, farmers expected an instant solution rather than a diagnostic process.

*I mean in Nigeria, you know, farmers, as veterinary doctors, farmers expect you to perform magic and instantaneous magics. (P.9) Farmer and Para-veterinarian*

In summary, participants described how insufficient knowledge of AMU and AMR impacted their attempts at prudent use of antibiotics, which was described as resulting from a lack of training and poor relationships between farmers and AHPs.

### 3.3. Theme 3: Taking Responsibility

The third theme related to the notion of responsibility. Most described how they felt that the government did not take enough responsibility for the misuse of antibiotics and needed to intervene. Some participants also felt that other people were not aware of AMR and did not use or prescribe AMU prudently and were therefore responsible for the problem of AMR. Some participants described how social responsibility was key to increasing awareness about the importance of prudent AMU.

#### 3.3.1. Government Responsibility

AHPs and other related key players described how the government needed to take more responsibility for addressing AMU and AMR through having rules and regulations, having capacity to enforce and enforcing these regulations.

*Most farms are not the place to start. Start with the government and also the veterinary body they are supposed to make sure that there are rules and regulations. (P.12) Agricultural ministry worker*

Furthermore, while there are regulatory agencies creating policies, participants described how such policies were not being enforced, leaving the animal health sector free to operate unregulated.

*I'll say generally in Nigeria it's not like there are not policies, but they are not actually being implemented. (P.12) Agricultural ministry worker*

Some participants also felt that the regulatory agencies were lacking the capacity to enforce the policies being created.

*OK. Like I was saying, I said in Nigeria, in Nigeria here there are regulatory agencies are not up to the task. (P.2) Veterinarian*

Furthermore, participants also described how medication was easy to access in the markets due to the lack of regulation as well as unlicensed and illegal production of antibiotics.

*Well, you know why you don't have when you don't have proper means of standardising drugs. Or you have illegal production everywhere. Everybody can just sit in your home and produce the drug and then people just take it and buy whatever they see. I have heard of a lot of people who actually is that easily accessible for them. (P.6) Veterinarian*

The access to antibiotics without prescription coupled with the lack of enforcement of regulation increases the risk of having poor AMU practices such as self-medication by farmers instead of consulting with AHPs.

*Because I don't think anything has been done because you can just go to a normal shop and see them selling drugs and people can just get drugs and nothing has been done. (P.12) Agricultural Ministry Worker*

#### 3.3.2. Personal Responsibility

Many participants also blamed individuals as well as the government. For example, they described how other farmers or other AHPs were not aware of AMR and did not use AMU prudently and were therefore responsible for the development of AMR. None, however, reflected on their own role in this or included themselves as bearing part of this responsibility.

*…some of the attitudes of, of our veterinarians and even the para-veterinarians that do treats. So it's not good at all because even then they need an orientation on how to use this antibiotics because they believe that whenever whichever case they came up with they cannot handle that case without antibiotics. So that the general mentality of our people here that any sickness you must use antibiotic before you can control it. (P.1) Veterinarian*

*I think farmers and other people who use antibiotics do not have sufficient knowledge about what antimicrobial resistance is all about. (P.3) Veterinarian*

Due to this lack of responsibility, from both the government and individuals, some participants suggested using social responsibility and focusing on the benefit to others and their health as a motivator for responsible AMU.

Highlighting farmer responsibility for reducing the risk of resistant infection transmission between birds and humans was also identified as a way for AHPs to intervene by emphasising to farmers the importance of prudent use of antibiotics on farms.

In summary, the third theme described a key role for acknowledging responsibility for appropriate antibiotic use and how this could be a barrier to developing AMR interventions. Many participants felt that the government should take more responsibility and enforce relevant regulations. The participants also pointed the finger of blame at others whom they saw as responsible for the misuse of antibiotics and the rise of AMR. Some participants also highlighted the role of social responsibility as a means of encouraging the use of AMR interventions.

## 4. Discussion

This thematic analysis aimed to understand the motivators and barriers to AMR intervention for poultry farmers and AHPs in Nigeria. Three themes were described in the data relating to (i) issues in access, (ii) lack of knowledge and (iii) taking responsibility.

The first theme ‘*issues of access*' described how prudent AMU and the uptake of interventions were limited due to a lack of access to factors such as time, money, laboratories and expertise. These findings align with general reports of barriers to good practice within the animal health sector. For example, time and money have previously been documented as lacking within the animal health sector, with farmers requiring more than one trade and therefore working alongside their farming jobs [16]. In SSA, 76% of those living in extreme poverty work in agriculture. Furthermore, of those working in agriculture, only just over half (50.4%) are not in poverty compared to 83.6% in nonagricultural sectors [16]. Requiring secondary jobs to stay afloat in agriculture is not limited to Nigeria but is also seen in other parts of the world, including the UK, the USA and India [17–19].

The impact of poor access to laboratories and expertise also aligns with previous research. For example, two cross-sectional surveys of AHPs in Nigeria found that 82% described the unavailability of laboratories as a barrier to performing antimicrobial susceptibility testing [20] and 32% of AHPs did not undertake antimicrobial susceptibility testing before prescribing antimicrobials [21]. This theme highlights how access issues, to a range of factors, interplay and are, for farmers and AHPs, barriers to engaging with and using AMR interventions. If these resources are not available for farmers and AHPs to access, it limits their ability to engage.

Furthermore, in terms of expertise, in 2020, there were only 9213 veterinarians registered in Nigeria (including those dead or no longer practising) [21]. This is approximately 0.04 per 1000 people [22], low compared to the numbers in the European Union, where there are 10 times as many, 0.42 veterinarians per 1000 people [23]. Overall, the issues of access were seen as critical to the prudent use of antimicrobials and the uptake of AMR interventions. Addressing this barrier is therefore needed to improve the use of AMR interventions.

The second theme described in the data is related to *‘lack of knowledge'* and highlights the lack of training and poor relationships between farmers and AHPs. As a result, farmers described relying on their own knowledge. This finding aligns with a survey conducted among Scottish veterinarians and farmers, which concluded that farmers rely on their own experience of AMU rather than veterinary advice [24]. The lack of training for farmers was particularly highlighted around withdrawal periods, reflecting evidence from other LMICs. For example, one cross-sectional study found that livestock and poultry farmers in Telangana state, India, had little knowledge of withdrawal periods [25].

The AHPs expressed interest in gaining knowledge about AMU and AMR, and most felt that it was important that farmers also did so. However, one AHP expressed that giving farmers more knowledge was dangerous as they would then use their own experience rather than consulting a veterinarian. An intermediate level of knowledge on a given subject has previously been associated with a high level of overconfidence and negative attitudes to science [26].

Ensuring that farmers have more knowledge about AMU and AMR could help mitigate the risk of negative attitudes towards science and other AMR interventions. The hesitancy by AHPs to provide farmers with knowledge on AMS could be due to poor relationships between AHPs and farmers. The farmer–AHP relationship is important within AMS. An improved relationship can lead to shared responsibility towards reducing AMU [27]. The farmer–veterinarian relationship has been identified, along with cooperative discussions and openness around cost, as the most important factor impacting farm biosecurity - another intervention within the animal health sector that can reduce AMU [28]. This theme highlights how a lack of knowledge, as a result of the lack of training on AMU and AMR, as well as poor relationships between farmers and AHPs, may prevent farmers and AHPs from engaging with AMR interventions.

The final theme *‘taking responsibility'* illustrates farmers' and AHPs' complex feelings about personal responsibility and others' responsibility towards prudent AMU and AMR, including the government. AHPs felt that the government needs to enforce regulations on good antimicrobial practices. A qualitative assessment of the National Action Plan for AMR based on the Tracking AMR Country Self-assessment Survey for Nigeria found that some improvements have been made in meeting the aims of the National Action Plan [29]. However, there are gaps, especially within the animal health sector [29]. The sentiment of lacking government responsibility is also prevalent in the human medical sector [30]. Up to 86.5% of antimicrobials are dispensed without a prescription in Nigeria [31]. Having rules and regulations on AMU and dispensing to motivate engagement with AMR interventions and good practice are important, but if there is no capacity to enforce these and they are not actually enforced, then the impact may not be realised.

However, it was not only the government that was seen in a negative light. A general feeling emerged that other farmers and AHPs were not prudent and responsible in their use of antimicrobials. However, no one acknowledged themselves as being part of the issue, implicitly implying that they were not contributing to AMR. This could be explained by the sociopsychological phenomenon ‘diffusion of responsibility'. Diffusion of responsibility indicates that people are less likely to take responsibility for their actions or inactions if there is a large group of people or witnesses [32]. As the poultry sector involves many farmers, and a large quantity of antimicrobials are used, no one farmer feels pressured to change their practices. Displacing responsibility may result in farmers' displacing the need to engage with AMR interventions.

Using social responsibility, which some AHPs spoke about using to implore the risk of AMR and the importance of prudent AMU, may be a way to combat this phenomenon. Social responsibility is the phenomenon of doing things that benefit the community or the collective rather than just oneself. Social responsibility has been shown to be effective in other public health intervention settings. In a Scottish study of smoke-free legislation, the concern of secondary smoke inhalation in nonsmokers was reported as a key factor influencing smokers to stop smoking [33]. This theme highlights the importance of establishing responsibility and ensuring that actors understand their responsibility. Using the technique of imploring social responsibility more widely may act as a motivator for farmers and AHPs to participate in AMR interventions.

Engaging poultry farmers and veterinarians in AMR interventions is a multifaceted issue that requires a multifaceted approach. An example of why a multifaceted approach is required is farmers' adherence to withdrawal periods. If information about the withdrawal period is not provided to farmers due to poor relations between farmers and AHPs, then farmers will not have the relevant knowledge to adhere to the withdrawal period. Even if farmers do have knowledge to adhere to withdrawal periods, the cost related to observing the withdrawal periods may push farmers to slaughter and sell birds during this period. In addition, the farmers may not feel responsible for driving AMR even if they are knowledgeable about withdrawal periods. This example illustrates that just mitigating one barrier or encouraging one motivator cannot stand alone in engaging farmers or AHPs in a change.

The strengths of this paper lie in its in-depth analysis and novel contribution to the understanding of motivators and barriers around AMR intervention in AHPs and poultry farmers in Nigeria. Nonetheless, there are some limitations. The interview language was English, the official language of Nigeria. However, this does not mean that everyone speaks English [34]. There is a range of dialects in Nigeria [35]. By interviewing in English, some participants may have expressed themselves differently than if they were interviewed in their primary language. The interviews were performed virtually. This allowed for interviews to take place across multiple states. However, a virtual interview may mean that rapport is not built with the interviewee in the same way. Participants may not share as much information as they would have done if a relationship was more established, such as in a face-to-face interview.

## 5. Conclusions

In conclusion, this study offers novel insight into the motivators and barriers surrounding the implementation of AMR interventions in the animal health sector in Nigeria. Engaging veterinarians and poultry farmers in AMR is a multifaceted issue that requires a multifaceted response. There is a current lack of access to money, time, laboratories and expertise. There was a general feeling that there was a lack of knowledge, originating from insufficient training and poor relationships between AHPs and farmers. Neither farmers nor AHPs felt personally responsible for the inappropriate AMU that occurred, but instead felt that others, including the government, were responsible for the current situation. Some participants highlighted how they used social responsibility to motivate farmers to use antimicrobials more prudently due to the lack of government responsibility around antimicrobials. While farmers and AHPs all had differing opinions about AMR interventions and the current situation in the animal health sector, there was an openness to interventions within the right settings. Future research could use the findings from these interviews to develop interventions that consider the factors surrounding access, knowledge and responsibility.

## Figures and Tables

**Table 1:** Participant groups.

Category	Subcategory
Farmer	Poultry (commercial layers, broilers or breeders)

Animal health professional (AHP)	Veterinarian (degree holder)
	Para-veterinarian (diploma or certificate holders)

Other related key player (ORKP)	Agriculture Ministry
	Feed processing companies
	Vet diagnostic laboratories
	Pharmaceutical industry (retailers)

## Data Availability

The data that support the findings of this study are available from the corresponding author upon reasonable request.

## Supporting Information

Supporting Data 1: Interview schedule for animal health professionals, farmers, and other related key players.

Supporting Data 2: Participant identification.
