# Supplementary material for: A Thematic Analysis of Motivators and Barriers to Antimicrobial Resistance Interventions With Farmers and Animal Health Professionals in Nigeria
Source: Vet Med Int. 2025 Oct 21;2025:8043291. doi: 10.1155/vmi/8043291 (PMC12567297; doi:10.1155/vmi/8043291)
Supplement: Supporting Information — Additional supporting information can be found online in the Supporting Information section. [file 8043291.f1.docx]

Supplementary Material

# *Supplementary Data 1*

**Interview Schedule for Animal Health Professionals**

| **Background**  Firstly I’d like to ask you some questions about your work and how you come into contact with antimicrobials through work | |
| --- | --- |
| 1. What is your background? | Prompts:  *What is your profession?*  *How long have you been working in this field?* |
| 1. Please tell me about the last time you prescribed antimicrobials for an animal/animals | If give a one-word answer “*Please could you explain why that is?”*  Prompts: *Did you base it on diagnostics? If yes, what?*  *Did you use antimicrobial guidelines? If yes, which?*  *What did the farmer think about your choice of treatment?* |
| 1. Over the last week when you’ve been called to treat animals. Have you found that farmers have treated animals with antimicrobials before calling you out? | Prompt: *What kind of antimicrobials have they used?*  Human antimicrobials? |

| **Issue Perception**  As you, (mentioned/may know) we become more and more aware of antimicrobial resistance. We are mainly interested in antibiotics a type of medicine that is essential to cure infections caused by bacteria. Over time antibiotics lose their effect as bacteria build up resistance. This happens faster when antibiotics are used incorrectly or more often than needed. Antibiotics are used incorrectly in both people and animals. Using them incorrectly in animals can cause resistance in both animals and people. I’d like to hear your options on this. | |
| --- | --- |
| 1. Do you talk to colleagues about antimicrobial use and antimicrobial resistance? | If yes: *What do you talk about?*  If no: *Do you discuss other animal health-related subjects with colleagues and if yes, what?*  Prompt: *Do your colleagues have different opinions to you?* |
| 1. Do you know any ways that practices around antimicrobials are being changed currently? And could you tell me about them? | Prompt:  *How do you feel about these changes?*  (Benefit/Not) (Acceptable not)  *Are they something you would be willing to do?* Why/Why Not?  **Explain AMS as needed** |
| 1. Would you do any of these things differently if you got to make the decision? | Prompt:  If yes/no *“Please could you explain why and what”* |

| **Future Changes**  *Thank you for telling me about the ways you see antimicrobial use being changed, I would like to ask you about your personal experience and if you* | |
| --- | --- |
| 1. Are there things that you think could change the way you interact with antimicrobials at work? | Prompt: education, guidelines, more information from AHPs (farmers), financial feasibility  *Thank you for your examples, would you be willing to adopt these things if they existed within your community?*  *Do you think they would be a benefit to you colleagues/community?* |
| 1. Are there any reasons that you do not do these things currently? | Prompts: Acceptability |
| 1. Are there any tools, information, or resources you think would help you work with antimicrobials in a way you mentioned/would like to? | Prompt: Relate prompts to interventions perceived as feasible |

| **Interventions**  Thank you for answering my questions so far, I would now like to ask you about some specific scenarios | |
| --- | --- |
| 1. How would you feel about training around dosage of antimicrobials? This would include dosage charts and visual cues. | Prompt: Acceptable?  *Would you be likely to do this? Why? Why not?*  *Do you think this would benefit you? Why? Why Not?* |
| 1. How would you feel about training in sanitation and disease control (for farmers)? | Prompt: Acceptable?  *Would you be likely to do this? Why? Why not?*  *Do you think this would benefit you? Why? Why Not?* |
| 1. How would you feel about participating in discussions about antimicrobial use, resistance, and its impact on animal and human health? | Prompt: Acceptable?  *Would you be likely to do this? Why? Why not?*  *Do you think this would benefit you? Why? Why Not?* |
| 1. How would you feel about training (for AHP) using guidelines for antimicrobial use and alternatives? | Prompt: Acceptable?  *Would you be likely to do this? Why? Why not?*  *Do you think this would benefit you? Why? Why Not?* |

**Interview Schedule for Farmers**

| **Background**  Firstly I’d like to ask you some questions about your work and how you come into contact with antimicrobials through work | |
| --- | --- |
| 1. What is your background? | Prompts:  *What is your profession?*  *How long have you been working in this field?* |
| 1. Please tell me about the last time you prescribed antimicrobials for an animal/animals | If give a one-word answer “*Please could you explain why that is?”*  Prompts: *Did you base it on diagnostics? If yes, what?*  *Did you use antimicrobial guidelines? If yes, which?*  *What did the farmer think about your choice of treatment?* |
| 1. Please tell me about the last time you used antimicrobials for your animals/one of your animals | Prompt:  *What symptoms do they have?*  *Do you do any testing before treating?* |
| 1. Where do you get the antimicrobials from when you treat your animals? | Prompt: Veterinarian, para-veterinarian, pharmacy, feed, other,  *Do you feed your animals feed with antimicrobials in them?* |

| **Issue Perception**  As you, (mentioned/may know) we become more and more aware of antimicrobial resistance. We are mainly interested in antibiotics a type of medicine that is essential to cure infections caused by bacteria. Over time antibiotics lose their effect as bacteria build up resistance. This happens faster when antibiotics are used incorrectly or more often than needed. Antibiotics are used incorrectly in both people and animals. Using them incorrectly in animals can cause resistance in both animals and people. I’d like to hear your options on this. | |
| --- | --- |
| 1. Do you talk to colleagues about antimicrobial use and antimicrobial resistance? | If yes: *What do you talk about?*  If no: *Do you discuss other animal health-related subjects with colleagues and if yes, what?*  Prompt: *Do your colleagues have different opinions to you?* |
| 1. Do you know any ways that practices around antimicrobials are being changed currently? And could you tell me about them? | Prompt:  *How do you feel about these changes?*  (Benefit/Not) (Acceptable not)  *Are they something you would be willing to do?* Why/Why Not?  **Explain AMS as needed** |
| 1. Would you do any of these things differently if you got to make the decision? | Prompt:  If yes/no *“Please could you explain why and what”* |

| **Future Changes**  *Thank you for telling me about the ways you see antimicrobial use being changed, I would like to ask you about your personal experience and if you* | |
| --- | --- |
| 1. Are there things that you think could change the way you interact with antimicrobials at work? | Prompt: education, guidelines, more information from AHPs (farmers), financial feasibility  *Thank you for your examples, would you be willing to adopt these things if they existed within your community?*  *Do you think they would be a benefit to you colleagues/community?* |
| 1. Are there any reasons that you do not do these things currently? | Prompts: Acceptability |
| 1. Are there any tools, information, or resources you think would help you work with antimicrobials in a way you mentioned/would like to? | Prompt: Relate prompts to interventions perceived as feasible |

| **Interventions**  Thank you for answering my questions so far, I would now like to ask you about some specific scenarios | |
| --- | --- |
| 1. How would you feel about training around dosage of antimicrobials? This would include dosage charts and visual cues. | Prompt: Acceptable?  *Would you be likely to do this? Why? Why not?*  *Do you think this would benefit you? Why? Why Not?*  Aim: Understand if this intervention is feasible, acceptability and benefit to the community |
| 1. How would you feel about training in sanitation and disease control (for farmers)? | Prompt: Acceptable?  *Would you be likely to do this? Why? Why not?*  *Do you think this would benefit you? Why? Why Not?*  Aim: Understand if this intervention is feasible, acceptability and beneficial to the community |
| 1. How would you feel about participating in discussions about antimicrobial use, resistance, and its impact on animal and human health? | Prompt: Acceptable?  *Would you be likely to do this? Why? Why not?*  *Do you think this would benefit you? Why? Why Not?*  Aim: Understand if this intervention is feasible, acceptability and benefit to the community |
| 1. How would you feel about training (for AHP) using guidelines for antimicrobial use and alternatives? | Prompt: Acceptable?  *Would you be likely to do this? Why? Why not?*  *Do you think this would benefit you? Why? Why Not?*  Aim: Understand if this intervention is feasible, acceptability and benefit to the community |

**Interview Schedule for Other Related Key Player**

| **Background**  Firstly I’d like to ask you some questions about your work and how you come into contact with antimicrobials through work | |
| --- | --- |
| 1. What is your background? | Prompts:  *What is your profession?*  *How long have you been working in this field?* |

| **Issue Perception**  As you, (mentioned/may know) we become more and more aware of antimicrobial resistance. We are mainly interested in antibiotics a type of medicine that is essential to cure infections caused by bacteria. Over time antibiotics lose their effect as bacteria build up resistance. This happens faster when antibiotics are used incorrectly or more often than needed. Antibiotics are used incorrectly in both people and animals. Using them incorrectly in animals can cause resistance in both animals and people. I’d like to hear your options on this. | |
| --- | --- |
| 1. How do you interact with antimicrobial resistance/antimicrobials at work? | Prompt:  Agriculture ministry: *Are you involved in creating or implementing law/policy that looks at antimicrobial resistance?*  Veterinary lab: *What kind of testing do you perform?* |
| 1. Do you talk to colleagues about antimicrobial use and antimicrobial resistance? | If yes: *What do you talk about?*  If no: *Do you discuss other animal health-related subjects with colleagues and if yes, what?*  Prompt: *Do your colleagues have different opinions to you?* |
| 1. Do you know any ways that practices around antimicrobials are being changed currently? And could you tell me about them? | Prompt:  *How do you feel about these changes?*  (Benefit/Not) (Acceptable not)  *Are they something you would be willing to do?* Why/Why Not?  **Explain AMS as needed** |
| 1. Would you do any of these things differently if you got to make the decision? | Prompt:  If yes/no *“Please could you explain why and what”* |

| **Future Changes**  *Thank you for telling me about the ways you see antimicrobial use being changed, I would like to ask you about your personal experience and if you* | |
| --- | --- |
| 1. Are there things that you think could change the way farmers/AHPs interact with antimicrobials at work? | Prompt: education, guidelines, accessibility, financial feasibility, acceptability, benefit |
| 1. Are there any tools, information, or resources you think change the way AHPs and farmers work with antimicrobials? | Prompt: Relate prompts to interventions perceived as feasible |

*Supplementary Data 2*

**Participant Identification**

| Participant Code | Participant ID | Participant Profession |
| --- | --- | --- |
| AHP-V-1 | P.1 | Animal Health Professional (Veterinarian) |
| AHP-V-2 | P.2 | Animal Health Professional (Veterinarian) |
| AHP-V-3 | P.3 | Animal Health Professional (Veterinarian) |
| AHP-V-4 | P.4 | Animal Health Professional (Veterinarian) |
| AHP-V-5 | P.5 | Animal Health Professional (Veterinarian) |
| AHP-V-6 | P.6 | Animal Health Professional (Veterinarian) |
| AHP-VFarmer-1 | P.7 | Poultry Farmer  Animal Health Professional (Veterinarian) |
| AHP-VFarmer-2 | P.8 | Poultry Farmer  Animal Health Professional (Veterinarian) |
| AHP-VPFarmer-1 | P.9 | Poultry Farmer  Animal Health Professional (Para-veterinarian) |
| Farmer-1 | P.10 | Poultry Farmer |
| Farmer-2 | P.11 | Poultry Farmer |
| ORKP-AM-1 | P.12 | Other related key player (Agricultural ministry) |
| ORKP-Farmer-1 | P.13 | Poultry Farmer  Other related key player |
| ORKP-Feed-FarmerAHP | P.14 | Poultry Farmer  Other related key player (Feed store) |
| ORKP-PI-1 | P.15 | Other related key player (Pharmaceutical Industry) |
| ORKP-PI-2 | P.16 | Other related key player (Pharmaceutical Industry) |
| ORKP-Research-1 | P.17 | Other related key player (Academic Research) |
| ORKP-Research-2 | P.18 | Other related key player (Academic Research) |
| ORKP-VL-1 | P.19 | Other related key player (Veterinary Lab Staff) |
| ORKP-VL-2 | P.20 | Other related key player (Veterinary Lab Staff) |
| ORKP-VL-3 | P.21 | Other related key player (Veterinary Lab Staff) |
| ORKP-VS-1 | P.22 | Other related key player (Veterinary Student) |
